# Maintenance Versus Transmission Deficits: The Effect of Delay on Naming Performance in Aphasia

**DOI:** 10.3389/fnhum.2019.00406

**Published:** 2019-11-27

**Authors:** Nadine Martin, Gary S. Dell

**Affiliations:** ^1^Department of Communication Sciences and Disorders, Temple University, Philadelphia, PA, United States; ^2^Beckman Institute and Department of Psychology, University of Illinois at Urbana–Champaign, Urbana, IL, United States

**Keywords:** short-term memory, naming, temporal processing, word retrieval, aphasia

## Abstract

We propose that deficits in lexical retrieval can involve difficulty in transmission of activation between processing levels, or difficulty in maintaining activation. In support, we present an investigation of picture naming by persons with aphasia in which the naming response is generated after a 1 s (sec) cue to respond in one condition or a 5 s cue to respond in another. Some individuals did better after 5 s, some did worse after 5 s, and some were not impacted by the delay. It is suggested that better performance after 5 s indicates a transmission deficit and that worse performance after 5 s indicates a maintenance deficit. To support this hypothesis, we adapted the two-step semantic-phonological model of lexical retrieval (Schwartz et al., 2006) so that it can simulate the passage of time and can simulate lesions in transmission (its semantic and phonological connection strength parameters) and/or maintenance (its decay parameter). The naming error patterns after 1 and 5 s for each participant were successfully fit to the model. Persons who did better after 5 s were found to have low connection strength parameters, persons who did worse after 5 s were simulated with an increased decay rate, and persons whose performance did not differ with delay were found to have lesions of both types. Some potential theoretical and clinical implications are discussed.

## Introduction

Aphasia, a language impairment that follows brain damage is accompanied by reduced verbal short-term memory (STM) capacity that is commensurate with the severity of the language impairment (Martin and Saffran, 1997; Martin and Ayala, 2004; Martin and Gupta, 2004). We attribute the association between aphasia and reduced verbal STM to a very old idea: a person’s ability to maintain the semantic or phonological representations of words depends on mechanisms that carry out the retrieval of these representations when speaking and listening. If one has difficulty producing and understanding a word, one will have trouble maintaining it. Although this claim is often made (Berndt and Mitchum, 1990; Saffran, 1990; Martin et al., 1996) and debated (Shelton et al., 1992; Martin and Freedman, 2001; Martin R.C., 2005), it has been difficult to specify with sufficient precision that it can be used to understand and remediate aphasia. In this paper, we describe a model of aphasia that may explain the production and short-term maintenance of single words and present data that test the model.

Computational models of language production and aphasia that are based on spreading activation (e.g., Dell et al., 1997, 2004; Foygel and Dell, 2000; Rapp and Goldrick, 2000; Ueno et al., 2011; Walker and Hickok, 2016) represent words and their sounds as a network of units connected through weighted links. Aphasic production deficits are viewed as a failure of spreading activation to activate the correct units, relative to incorrect ones, explaining the nature and frequencies of paraphasias that occur in picture naming or word repetition tasks. These models attribute aphasia to either a transmission failure (e.g., weak or noisy connection weights) or a failure to maintain activation of a unit (e.g., overly fast decay of activation). In our work, we have used both accounts to simulate aphasia, and particularly to simulate individual persons with aphasia as opposed to aphasic syndromes (Martin et al., 1994, 1996; Schwartz et al., 2006; Dell et al., 2007, 2013; Nozari et al., 2010). It turns out, though, that transmission and maintenance failures are difficult to distinguish. One can fail to activate the/k/of the target “cat” because connections to it are weak, or because its activation decays away.

Although current models have been able to distinguish the “*where*” of a deficit (e.g., lexical-semantic vs. lexical-phonological), they are less able to distinguish deficit “*mechanisms*,” e.g., transmission vs. maintenance (Foygel and Dell, 2000). There are two reasons for this. First, most data on lexical deficits in aphasia come from production tasks that do not manipulate or measure the temporal dynamics of production. Second, the models themselves make no claims about the passage of time and its effects on accuracy of word retrieval. An exception to these generalizations involved early studies that found that the rare semantic errors made in word repetition tasks by persons with aphasia can be promoted when there is more time before the response (e.g., Martin et al., 1994, 1996). In recent work, we have investigated the effects of time passage on word retrieval and we proposed that such errors are caused specifically by an overly strong decay of activation.

We have investigated the effects of time passage on word retrieval by adding a temporal component (response delay) to word retrieval tasks (Martin et al., 1996, 2018; Martin and Dell, 2017). These studies have revealed some intriguing findings that we investigate further in this study: some aphasic individuals perform more poorly after a time delay while others benefit from additional time to respond. Here, we provide some data from the Temple Assessment of Language and Short-term memory in Aphasia (TALSA; Martin et al., 2018) that demonstrate the change in accuracy of naming following a response delay and (2) test the hypothesis that better or worse performance on delayed naming tasks maps onto deficits of transmission or maintenance, respectively. To test this claim, we created a new version of the model of word production, the Semantic-Phonological Model (SP), which has been used in many of our studies of word production, but most recently in a study of Dell et al.’s (2013) that identified the neural correlates of semantic and phonological components of word processing.

### The Present Study

For this study, we adapted the SP model to better represent the passage of time and treated both connection strength values and decay rate as lesionable parameters. Both of these alterations were necessary to apply the model to data showing changes in accuracy of word production after a 5 s response delay. We demonstrate that reduced connection strength can account for performance that improves after 5 s and increased decay rate can explain worse performance after a response delay. We also show that the new SP model, which we call the “slow” SP-decay model, can directly fit the error proportions in naming that occur after different response delays, including worse performance, better performance and no change in accuracy levels. Also, as in our previous modeling work, the goal is not just to model overall correctness, but also the proportions of the error types, such as non-word errors and various kinds of lexical errors.

The first part of the study is empirical. We sought behavioral evidence for temporal dimensions of impairment in lexical processing by evaluating the picture naming performance of individuals with chronic aphasia. We administered the picture naming test under two response delay conditions (1 and 5 s), allowing us to observe the effects of a time delay on accuracy. Based on a prior study (Martin and Dell, 2017), we expected to find a few individuals that were worse after a 5 s response delay, while for others, accuracy would increase after a 5 s response delay. We also expected many to show little difference, or at least differences that are not easily detectable.

The second part of the study introduces the slow SP model. Unlike most previous versions of the model, it represents the passage of time so that response delays can be modeled and includes decay as a lesionable parameter. The expectation is that the naming error pattern made by individuals whose performance is worse after a 5 s delay could be characterized by a weak (larger) decay parameter, and individuals whose naming benefits from the extra time in the 5 s delay could instead be fit by assuming weak (lower) connection strength parameters. The data and model fitting potentially have both theoretical and clinical implications. They can test the temporal assumptions of the model and can identify deficits and potential treatments related to those aspects.

**Part 1. The effects of response delay on accuracy of picture naming in people with aphasia**.

## Materials and Methods

### Participants

#### Participants With Aphasia

The 90-item picture naming subtest of the Temple Assessment of Language and Short-term Memory in Aphasia (TALSA) was administered in two different time periods, with slight differences in the administration format, but no difference in the item content (see details in description below). In the most recent administration of the test (2015–2018), 24 people with chronic aphasia completed the TALSA naming test. In an earlier administration period (2008–2012), 21 people with chronic aphasia completed the test but six of these individuals were among those who were tested in the 2015–2018 period. For these six, we used the data sets from the most recent testing period. Thus, there were 39 participants (15 from the early testing period and 24 from the recent one). The classical aphasia types represented in this group included, Broca, Wernicke, Conduction, Anomia, and Transcortical Motor. Participants with aphasia were at least 6 months post-onset and had single or multiple left-hemisphere lesions resulting from a cerebrovascular accident (CVA).

Table 1 shows the etiologies of aphasia, months post-onset at the time of testing, the aphasia quotient from the Western Aphasia Battery-Revised (Kertesz, 2006) and the period in which they were tests (2008–2012 or 2015–2018). There were 15 female and 24 males in the sample. Average age was 57 years [standard deviation (SD): 9.48 range: 32–78]. The average number of months post-onset at time of testing was 82 months (*SD*: 77.79) and ranged from 6 to 333 months. All but one of the participants were high school educated, and the years of education ranged from 7 to 19 years, with an average of 14 years (*SD*: 2.57).

**TABLE 1:** Information on (1) etiology of stroke, time post-onset and aphasia severity and (2) period when tested and inclusion of the participant’s data in the naming analysis.

**Participant ID**	**Time post-onset (months)**	**Etiology**	**WAB-R AQ^1^**	**Period tested**	**Qualified for naming analysis**
FS1	11	LCVA^2^, interval resorption of hemorrhage within the left temporal lobe.	76.4	2008–2011	Y
SX3	291	LCVA, hemorrhagic infarct in the left basal ganglia.	92.8	2015–2018	Y
KC3	155	LCVA involving the middle and superior temporal gyrus, middle and inferior frontal gyrus, inferior parietal lobe (supramarginal and angular gyrus), and extending down to the lateral ventricle with damage to the basal ganglia. Temporal pole is preserved.	64.1	2015–2018	Y
EH4	123	LCVA, left hemisphere stroke with damage to the insular cortex, middle and inferior frontal gyrus extending to the parietal lobe. Temporal lobe in intact.	81.4	2015–2018	Y
CM5	82	LCVA, left parietal infarct with stable petechial hemorrhages of the bilateral centrum semiovale, cerebellum and brainstem.	70.0	2015–2018	N
DD6	78	LCVA, affecting left frontal parietal regions	55.6	2008–2011	Y
CT7	133	LCVA, left middle cerebral artery (MCA) infarct with extension into the posterior limb of the left internal capsule.	33.6	2008–2011	Y
MI10	86	LCVA	71.5	2008–2011	Y
EC15	103	LCVA	84.9	2008–2011	Y
TB16	69	LCVA, LMCA affecting watershed areas of LMCA/posterior cerebral artery (PCA) with hemorrhagic transformation	55.6	2008–2011	N
IU19	12	LCVA	82.0	2008–2011	N
SL21	106	Left CVA (parietal aneurysm).	89.0	2008–2011	Y
QH22	22	LCVA, left transverse and sigmoid cerebral sinus thrombosis with secondary bleeding into the ischemic zone; subsequent left hemicraniectomy with evacuation of intracranial hemorrhage on the left side.	84.9	2008–2011	Y
EC25	333	LCVA, left frontoparietal craniotomy and clipping of left PCA aneurysm.	62.5	2015–2018	Y
KL27	37	LCVA, thalamic CVA	83.5	2008–2011	N
HI 28	21	Left occipital lobe infarct with several smaller satellite infarcts surrounding the posterior horn of the left lateral ventricle with several small acute infarcts within the left centrum semiovale and corona radiata; old right corona radiata and left sub-insular lacunar infarcts	65.3	2015–2018	Y
UN29	12	LCVA, left MCA, edema posterior aspect of left frontal lobe/left temporal parietal region with subacute petechial hemorrhage left basal ganglia and increased edema left caudate nucleus and left internal capsule consistent with evolving infarct.	33.8	2008–2011	Y
QC30	79	Left parietal infarct (non-hemorrhagic); chronic infarcts affecting left periventricular region, right corona radiata, bilateral basal ganglia and bilateral thalami.	93.2	2008–2011	N
SC32	14	LCVA	N/A	2008–2011	N
KU33	6	LCVA, left posterior temporal occipital lobe infarct.	90.5	2008–2011	Y
LT34	12	LCVA, left MCA occlusion involving the posterior left temporal lobe and left parietal lobe.	86.6	2008–2011	N
UP35	77	LCVA affecting the posterior 2/3s of the inferior frontal gyrus, subcortical white matter beneath the middle and superior frontal gyri, and the anterior superior insula cortex. The temporal lobe was intact.	88.6	2015–2018	N
DC37	23	LCVA, extensive left ACA, MCA and PCA infarctions. Extensive left craniotomy with stable subdural hemorrhage.	92.8	2015–2018	Y
KM38	224	LCVA, infarct affecting LMCA territory and portion of the LACA territory; extensive damage to frontal portions of the temporal and parietal lobes, down to lateral ventricles, sparing superior middle and frontal lobes. Insula and basal ganglia are severely damaged.	80.3	2015–2018	Y
CN39	27	LCVA, LMCA aneurysm and left internal carotid artery (ICA) occlusion, affecting left insular cortex, posterior 2/3s of the inferior frontal gyrus, inferior middle frontal gyrus, anterior margin of the angular gyrus and inferior insula. Temporal lobe is intact.	76.3	2015–2018	Y
HE41	71	LCVA, aneurysm in the region of the left MCA bifurcation/trifurcation, large hypoattenuating lesions within the left temporal, frontal, and parietal lobes into the frontoparietal lobes.	74.8	2015–2018	Y
DC44	56: 5	LCVA, chronic infarct in left basal ganglia extending into left periventricular frontal white matter with mild to moderate chronic ischemic white matter changes in right thalamus with left Wallerian degeneration.	93.5	2015–2018	N
XH46	23	LCVA, LMCA infarct with damage to the middle and inferior frontal gyrus and anterior insula. Some damage extending to the basal ganglia (head of the caudate).	73.1	2015–2018	Y
KG47	159	LCVA, infarct affecting territory of LMCA and LPCA including left parietal lobe and extending posteriorly to the left occipital lobe. There are tiny areas of acute ischemia in the cerebellar hemispheres, along the right parietal convexity and in the body of the corpus callosum. The insula and superior temporal gyrus anterior to temporoparietal junction are spared, but posterior white matter in the superior temporal gyrus is affected.	99.7	2015–2018	Y
UM48	44	LCVA, LMCA infarct affecting superior insula posteriorly, anterior parietal and posterior frontal lobes, as well as posterior middle and posterior inferior frontal gyrus. Indications of white matter ischemia.	89.2	2015–2018	N
KT53	25	LCVA, large left hemisphere lesion affecting posterior superior and posterior middle temporal gyri, with bulk of damage to inferior parietal lobule. Indications of posterior branch of MCA infarct. Medial parietal lobe is spared.	48.8	2015–2018	Y
NF54	31	LCVA, LMCA including the opercular region and extending posteriorly in the temporal lobe along the optic radiations	89.1	2015–2018	N
KK55	129	LCVA, moderate left frontal and temporal parietal infarct – left MCA distribution; hemorrhage medial to the left temporal region extending partially into left lenticular nucleus.	78.7	2015–2018	Y
MN56	114	LCVA, left temporoparietal region of hypodensity and sulcal effacement consistent with late acute/early subacute left MCA infarct.	81.1	2015–2018	N
BQ58	65	LCVA, large hypoattenuating lesions within the left temporal, frontal, and parietal lobes into the cortex of the frontoparietal lobes.	33.6	2015–2018	Y
BC60	25	Left anterior MCA distribution infarct involving the frontal and insular lobes, mild surface hypodensity may represent thrombosed MCA branches or petechial hemorrhages. Old tiny lacunar infarct in L caudate head.	71.4	2015–2018	Y
KG62	111	Large infarction left MCA distribution with small focal areas of hemorrhage; also complete occlusion of the left MCA secondary to thrombus.	66.3	2015–2018	Y
CI63	187	LCVA, left frontal MCA territory infarct and older left insular/frontoparietal infarct. Regions affected include posterior temporal lobe, inferior frontal gyrus, and insula. Most of the lesion is subcortical, with white matter projections from anterior temporal lobe interrupted. Subcortical structures indicate Wallerian degeneration and substantial deep white matter loss. Temporal pole is relatively preserved.	61.4	2015–2018	Y
DS68	13	LCVA, left insular Infarct and middle temporal lobe. Primary cortical damage is to the posterior insula, affecting white matter including the arcuate fasciculus. White matter damage extends from the posterior third ventricle to the anterior portion of the lateral ventricle.	82.7	2015–2018	Y

*^1^WAB-R = Western Aphasia Battery-R (Kertesz, 2006). ^2^LCVA, left cerebral vascular accident.*

All participants were administered the Western Aphasia Battery-Revised (Kertesz, 2006). This standardized screening test for language abilities in aphasia assesses language abilities such as naming, repetition and comprehension. It yields an Aphasia Quotient summarizing the overall language ability with a score between 0 and 100. The average Aphasia Quotient for this sample of people with aphasia (*n* = 39) was 75 (*SD*: 17.10) and scores ranged from 33.8 to 100.

#### Control Participants

Eleven individuals without aphasia or brain damage completed the same TALSA picture naming test that the persons with aphasia did. There were three males and eight females with an average age of 66.43 years (*SD*: 10.1 range: 47–80). Years of education ranged from 12 to 20 with an average of 15.57 years (*SD*: 2.80).

All participants voluntarily enrolled in this research program and signed a consent form approved by the Internal Review Board at Temple University.

### Materials

#### Naming Test

All participants completed the TALSAs 90-item picture naming (Martin et al., 2018). Picture names were 1–3 syllables in length. We used frequency ratings from Pastizzo and Carbone (2007) and divided the stimuli into high frequency (>25 occurrences per million, range 27 to 673) and low frequency (<25 occurrences per million).

#### Administration of the Naming Test

As noted above, we administered two versions of the test that varied in the format of administration, but not in content. The pictures and target names were identical in both versions of the test. In the first version, administered between 2008 and 2012, the 90 picture items were divided into three sets of 30 items. Each set was assigned to one of three response delay conditions, 1 s unfilled, 5 s unfilled and 5 s filled delay. Syllable length and word frequency were balanced across all three sets. After each of the three sets was administered in one of the three response delay conditions, they were then administered a second and third time (in separate testing sessions) in the other two response delay conditions. Thus, all 90 stimuli were administered in all three response delay conditions. For this study, we report only the data from the first two conditions, 1 s unfilled and 5 s unfilled, as we were interested in the effects of time, but not interference.

In 2015, we revised the administration of the test, presenting the 90 picture items blocked in a single response delay condition (e.g.,1 s unfilled response delay) and then in separate sessions, administering the same 90 items (randomized order) in the other two response delay conditions. The order of administering the test in the three response delay conditions was randomized across participants. Again, only the data from the unfilled conditions are reported in this study.

#### Testing Procedure

For both versions, pictures were presented on a computer via e-prime software (Psychology Software Tools Inc., 2012) for 4 s, with a beep cue to name the picture 1 s (1-sec) or 5 s (5-sec) after it went off the screen. The next picture was presented 4 s after this cue and hence the participant had to respond within this 4 s period.

#### Scoring Procedures

Scoring and response categorization followed the guidelines of the Philadelphia Naming Test (Roach et al., 1996; Dell et al., 1997). The first complete response was counted as the response of interest. This is the first naming attempt with minimally a consonant-vowel or vowel-consonant (with schwa not being counted as a vowel). Attempts should not be self-interrupted, have a clear downward or upward intonation and is followed by a distinct pause.

#### Reliability of Scoring

Naming responses were transcribed by two research speech-language pathologists in the Aphasia Rehabilitation Research Laboratory where the testing took place.

Inter-rater reliability of scoring was evaluated using Cohen’s Kappa statistic on a random selection of participants (8 test administrations) which accounted for 15% of the data from the 2015–2018 sample. There was substantial agreement between the two scorers, *k* = 0.774 (*p* < 0.000) (Landis and Koch, 1977).

## Results

### Control Participants

For the 11 control participants the average score on the 1 s unfilled response delay condition was 0.98 (*SD*: 0.02, range: 0.94–1.00). On the 5 s unfilled response delay condition, the average score was 0.98 (*SD*: 0.02, range: 0.93–1.00).

### Participants With Aphasia

From the 39 sets of data collected during the two testing periods, we removed 12 whose correct naming proportion on the both the 1 and 5 s response delay conditions was greater than 0.90 correct. The remaining 27 sets of data were further analyzed to identify significant increases or decreases in accuracy following a 5 s response delay, in comparison to the 1 s response delay condition. The final column of Table 1 indicates whether a participant’s data was included in this further analysis (Y) or not (N).

Table 2 shows the proportions correct, the difference between proportions correct as a function of delay, and the significance and effect size (Cohen’s D) of those differences. Six participants (22%) demonstrated a significant change in accuracy after a 5 s response delay. Three showed better performance after 5 s (KG47, CI63, and KC3) and three showed worse performance (DS68, SL21, and UN29).

**TABLE 2:** Performance on the TALSA naming test (*n* = 90) with two testing conditions: 1- and 5-s response delay and test of the difference between these conditions.

	**Response delay**						
						
**Participant ID**	**1-s**	**5-s**	**5-s minus 1-s**	**z Statistic**	***p* Value**	**ln odds ratio**	**Cohen’s D**
DS68	0.77	0.53	–0.23	3.23	0.00	1.06	0.58
KG47	0.84	0.97	0.12	2.56	0.01	–1.68	–0.92
CI63	0.57	0.73	0.16	2.33	0.02	–0.74	–0.41
SL21	0.90	0.78	–0.12	2.18	0.03	0.94	0.52
KC3	0.67	0.80	0.13	2.01	0.04	–0.69	–0.38
UN29	0.10	0.02	0.08	1.99	0.05	1.59	0.87
KU33	0.67	0.79	–0.12	1.83	0.07	–0.63	–0.34
FS1	0.88	0.96	–0.08	1.81	0.07	–1.10	–0.60
XH46	0.52	0.64	–0.12	1.66	0.10	–0.51	–0.28
MI10	0.66	0.77	–0.11	1.64	0.10	–0.55	–0.30
EH4	0.83	0.73	0.10	1.62	0.11	0.60	0.33
KG62	0.52	0.63	–0.11	1.51	0.13	–0.46	–0.25
CN39	0.76	0.84	–0.09	1.48	0.14	–0.56	–0.31
QH22	0.81	0.88	–0.07	1.23	0.22	–0.51	–0.28
KT53	0.36	0.42	–0.07	0.92	0.36	–0.28	–0.15
EC15	0.87	0.83	0.04	0.63	0.53	0.26	0.14
MT50	0.68	0.71	–0.03	0.49	0.63	–0.16	–0.09
SX3	0.83	0.81	0.02	0.39	0.70	0.15	0.08
DC37	0.63	0.65	–0.02	0.31	0.76	–0.10	–0.05
BQ58	0.89	0.90	–0.01	0.24	0.81	–0.12	–0.06
HE41	0.83	0.82	0.01	0.20	0.84	0.08	0.04
HI28	0.81	0.80	0.01	0.19	0.85	0.07	0.04
CT7	0.74	0.73	0.01	0.17	0.87	0.06	0.03
KM38	0.71	0.70	0.01	0.16	0.87	0.05	0.03
DD6	0.66	0.64	0.02	0.16	0.88	0.05	0.03
EC25	0.68	0.68	0.00	0.00	1.00	0.00	0.00
KK55	0.88	0.88	0.00	0.00	1.00	0.00	0.00

Before we turn to modeling these data, it is useful to consider whether there are true differences due to delay in the sample, and whether these cases are possible examples of such differences. After all, in a sample of 27 individuals, one would expect one or two of them to be associated with a significant effect of delay by chance even if the manipulation had no true effect. The fact that there were six significant cases is somewhat reassuring. Perhaps more important is the sizes of the effects obtained, as measured by Cohen’s D, for example, for DS68, *D* = 0.58, for KG47, *D* = −0.92, for SL21, *D* = 0.52 and UN29, *D* = 0.87. (A positive value indicates worse performance on the 1 s delay.) With 0.80 considered to be a large effect and 0.50, a medium effect, the effect sizes support the legitimacy of these differences. Although we cannot be certain that we have identified just those individuals whose naming is affected by the delay, we are reasonably confident that such people exist and that the set of six that we have selected includes some.

In the next study, we will use the new version of the model to test the hypothesis that better performance after a delay arises from a transmission deficit (low connection strengths) and worse performance arises from a maintenance deficit (increased decay). We also will test whether the model can in general fit the response patterns. Finally we will model data from three participants whose data showed little or no difference in accuracy in the 1 and 5 s response delay conditions, to show that such cases are also consistent with the model.

### Preparation of the Data for Modeling

As we explain below, the output of the interactive two-step model includes correct responses and five categories of errors: Semantic, Formal, Mixed, Unrelated, and Non-word errors. For the most part, responses on this test by people with and without aphasia fall into these categories. However, some responses fall into categories not produced in this model and some fall into the category of “Other” (e.g., naming just a part of the picture, man → shirt). When an “Other” error is made, that test item is removed from the total number of items tested. Two response types that are not produced by the model are ‘No Reponses’ (saying nothing or otherwise reporting failure, e.g., “can’t”) and ‘Descriptions’ (providing a description of the portrayed object, e.g., “Some kind of animal, I think”). These responses are not removed from the analysis, but rather are distributed across the five model error types, in proportion to how frequently each of those error types occurs with that individual. Thus, this treatment does not change the proportion correct, nor does it change the relative proportions of the error types. The six sets of response distributions that will be modeled are presented in Table 3.

**TABLE 3:** Participants with significant change in accuracy on picture naming test after a 5 s response delay: distributions of responses (proportions) after 1 and 5 s response delays.

**Participant**	**Response delay**	***N***	**Correct**	**Semantic**	**Formal**	**Mixed**	**Unrelated**	**Non-word**
KC3	1 s	*n* = 86	0.67	0.13	0.01	0.00	0.00	0.14
	5 s	*n* = 89	0.80	0.14	0.00	0.04	0.00	0.00
CI63	1 s	*n* = 90	0.56	0.07	0.03	0.00	0.01	0.34
	5 s	*n* = 89	0.73	0.01	0.03	0.00	0.00	0.21
KG47	1 s	*n* = 88	0.84	0.13	0.00	0.00	0.00	0.00
	5 s	*n* = 87	0.97	0.00	0.00	0.00	0.00	0.00
SL21	1 s	*n* = 89	0.91	0.02	0.00	0.01	0.00	0.06
	5 s	*n* = 85	0.82	0.01	0.00	0.03	0.01	0.16
UN29	1 s	*n* = 90	0.10	0.03	0.03	0.00	0.28	0.59
	5 s	*n* = 88	0.02	0.02	0.10	0.00	0.40	0.33
DS68	1 s	*n* = 90	0.77	0.08	0.02	0.00	0.00	0.13
	5 s	*n* = 90	0.53	0.18	0.00	0.00	0.00	0.29

**Part 2. Computational study. Modeling the transmission and maintenance deficits in naming.**

**FIGURE 1:**
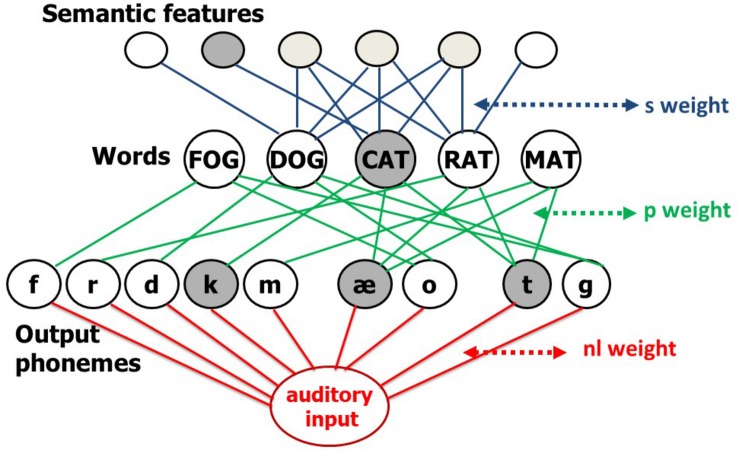
The interactive two-step model of lexical access. The s (semantic) connections (blue) transmit activation between semantic and word nodes, the phonological (p) connections (green) do the same between word and output-phoneme nodes. The red part of the network added a non-lexical (nl) route to support non-word and word repetition. The slow SP model that is implemented in the current paper does not include the non-lexical route.

The data from the naming study indicated two patterns of change in naming accuracy (better or worse) following a response delay. Here, we use the interactive two-step Semantic-Phonological (SP) model of word processing, to account for these patterns. The SP model of word retrieval consists of an interconnected network of semantic, lexical, and output phonological units, and a further set of connections between auditorily presented verbal input and the output phonological units (Figure 1). All connections are bidirectional, thus making the model’s flow of activation interactive. In naming, lexical access starts with a jolt of activation to the target word’s semantic features and then flows through the network. The activation function is linear with a decay component. Specifically, activation of a unit at a time step is equal to a fraction of its activation at the previous time step (the lost activation determined by the decay rate) plus any new activation delivered by its activated neighbors through weighted connections. Also, during each time step, a unit’s activation is perturbed by a normally distributed value with mean zero, and a standard deviation that is a linear function of the unit’s current activation (with a non-zero intercept). More activated units are noisier, but even units with no activation experience some noise. After a fixed number of time steps for activation to spread, the most active word unit of the appropriate grammatical category is selected, completing the first “step” of lexical access. Errors at this step are lexical (e.g., semantic, CAT→DOG; unrelated, CAT→LOG; formal, CAT→MAT, or mixed semantic-formal, CAT→RAT). A jolt of activation to the selected word unit initiates the second step. Activation then spreads throughout the network again for a fixed number of time steps, culminating in the selection of the most activated phonological units. Errors at this step are typically non-words (e.g., CAT→ “cag”) but can also be formally related to the target word (e.g., CAT→“mat”). Errors occur because of noise and spreading activation, which activates units other than the target units. Please see Schwartz et al. (2006) for details.

The model has successfully simulated patterns of error by speakers with aphasia in: (1) *Naming*, by assuming there are weak connections between semantic and word units (parameter s) or word and phonological units (parameter p) (Schwartz et al., 2006) and (2) *Word and non-word repetition* (Dell et al., 2007; Nozari et al., 2010), by including a mechanism that allows for production of phonological sequences that are not already stored in the lexicon (Hanley et al., 2004). This non-lexical route (Figure 1) lies in the connections between auditory input and output phonological units, and this connection strength is the parameter *nl*. Word repetition may involve both the non-lexical route and the lexical route corresponding to the second step of lexical access from meaning. To repeat a word, the model starts with a jolt of activation to the word unit and, for some individuals (see Nozari and Dell, 2013), a secondary jolt to the non-lexical route input unit.

The need to separate the s and p parameters is apparent from the error patterns of many of the persons with aphasia (e.g., Schwartz et al., 2006). A pattern with many non-word and formal errors, but few semantic errors suggests a low value of p, whereas a pattern with no non-word errors, but many lexical errors points to a low value of s. Also, it turns out that word repetition ability depends heavily on the value of p, and not on the value of s (Dell et al., 2013).

The current form of the SP model cannot be applied to the naming data obtained under different delays. In the model, activation spreads for a short and fixed period, essentially spreading all at once. Hence, there is no mechanism to explain how time affects processing. Consequently, we created the slow version of the naming model (without the non-lexical route) in which activation levels change more slowly and can be tracked over many time steps. We did this simply by reducing the amount of activation that spreads in each time step and the amount of decay that each unit undergoes in each step.

In the original model, normal performance was achieved with the s and p connection weights at 0.04 and with decay at 0.6. This yields a naming pattern of 97% correct, 2% semantic errors, and1% mixed errors. Changing s and p to 0.0003 and decay to 0.001 and leaving other model properties unchanged creates a very similar model, except that activation patterns take more time to develop.^1^

### Modeling Normal and Impaired Performance

Table 4 shows the simulation of normal performance and compares this to a lesion in the connection weights (parameters s and p), which reduces the transmission of activation in the network. Using the slow model, normal performance (97% correct) was simulated after between 8 and 20 time steps. Importantly, if we create a lesion in the weight parameters (reducing s and p to 0.0001), the model’s accuracy is low, but improves with time. After 8 time steps, performance is poor (47% correct) but improves when more time passes (e.g., 65% at time step 25).

**TABLE 4:** Slow version of interactive activation model: proportion of naming responses correct at each time step in the SP model under two connection weight conditions.

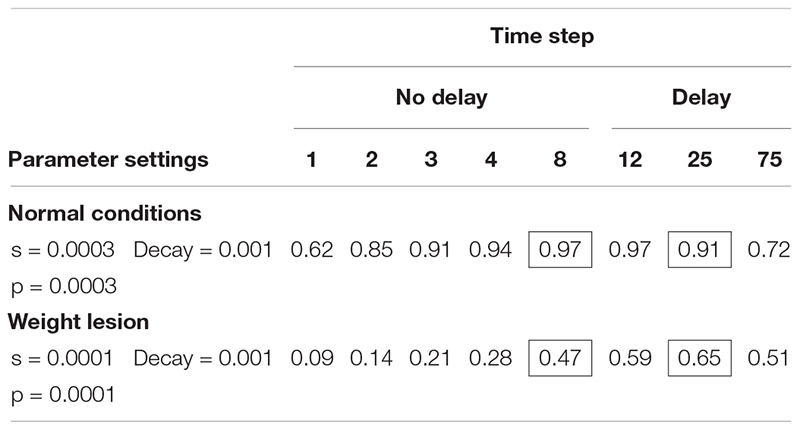

### Modeling the Pattern of Naming That Improves After a 5 s Response Delay

We then used the slow model to simulate the naming performance of the three people from the behavioral study reported in Part 1 who showed significant improvement in naming following a 5 s response delay, KC3, KG47, CI63. These data are shown in Table 5 and include the proportion of correct and erroneous naming responses produced by each participant when naming was delayed by 1 and 5 s. Below those data are the proportions of correct and erroneous naming responses produced by the model after 8 time steps and after 25 time steps and the parameters used to fit the model to the naming pattern. We used 8 and 25 time steps to simulate 1- and 5-s response delays, respectively. We assumed that 1-s corresponds to 5 time steps and thus the 5-s delay corresponds to 25 steps. But at the 1 s delay, the actual naming response typically occurred a bit later than 1 s on average. Hence, we assumed eight steps for this delay.

**TABLE 5:** Modeling the pattern of better naming after a response delay.

				**Response types**						
					
**Participant/model**	**Model parameters**	**Response delay/time steps**	***N***	**Correct**	**Semantic**	**Formal**	**Mixed**	**Unrelated**	**Non-word**	**RMSD**
KC3		1-s delay	*n* = 86	0.67	0.13	0.01	0.00	0.00	0.14	
Model	s^1^ = 0.00009	8 time steps		0.67	0.10	0.08	0.02	0.06	0.08	*0.047*
KC3	p^2^ = 0.0002	5-s delay	*n* = 89	0.80	0.14	0.00	0.04	0.00	0.00	
Model	DR^3^ = 0.001	25 time steps		0.79	0.08	0.04	0.02	0.02	0.05	*0.038*

KG47		1-s delay	*n* = 88	0.84	0.13	0.00	0.00	0.00	0.00	
Model	s = 0.00011	8 time steps		0.82	0.09	0.04	0.02	0.04	0.00	*0.031*
KG47	p = 0.00045	5-s delay	*n* = 87	0.97	0.00	0.00	0.00	0.00	0.00	
Model	DR = 0.001	25 time steps		0.88	0.07	0.01	0.02	0.01	0.00	*0.047*

CI63		1-s delay	*n* = 90	0.56	0.08	0.03	0	0	0.33	
Model	s = 0.000019	8 time steps		0.57	0.02	0.08	0.01	0.01	0.31	*0.035*
CI63	p = 0.000109	5-s delay	*n* = 89	0.73	0.01	0.03	0.00	0.00	0.22	
Model	DR = 0.001	25 time steps		0.71	0.04	0.05	0.01	0.01	0.18	*0.024*

*^1^s = semantic weight parameter. ^2^p = phonological weight parameter. ^3^DR = decay rate parameter.*

The fitting process was informal, as our goal was only to establish whether the model’s lesions can in principle create the kinds of differences that we see. We simply tried values of the s and p parameters that yielded performance in the range of each participant. For each case, the model captures the increase in accuracy after 5 s and also the changes in rates of different error types, especially a reduction in the non-word errors. To quantify the degree of fit, Table 5 shows the uncorrected root mean squared deviations (RMSDs) between the model and participant response-category proportions. The RMSD is calculated using the six proportions of each delay and thus there is a separate RMSD determined for the 1 s data and for the 5 s data.

### Modeling the Pattern of Naming That Becomes Worse After a 5 s Response Delay

Our next aim was to determine whether the slow SP model can account for the pattern of naming responses in which performance is worse after a 5 s response delay. It turns out that the slow version of the model cannot simulate poorer performance after a delay if the possible lesions are restricted to the s and p parameters. What is needed is a postulation of a decay rate deficit as opposed to a deficit of connection strength. When a decay rate lesion is applied to the slow model (Table 6) and the s and p weight parameters are held constant, performance is better with no delay (94%) than at the delay (44% at step 25). In this way, the slow SP-decay model may explain the patient differences; in one case there is weakness in information transmission, in the other, there is a weakness in maintenance.

**TABLE 6:** Slow version of interactive activation model: proportion of naming responses correct at each time step in the SP model, comparing connection weight, and decay lesions.

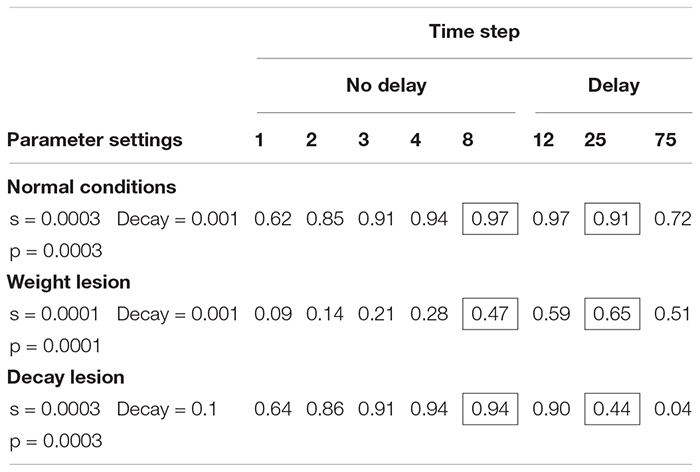

We used this model to simulate the naming performance of the three people from the behavioral study whose naming was significantly worse when a response was delayed by 5 s (SL21, UN29, and DS68, Table 7). The model captures the decrease in accuracy after 5 s as well as aspects of the changes in error patterns, particularly the increase in non-word errors.

**TABLE 7:** Modeling the pattern of worse naming after a response delay.

				**Response types**						
					
**Participant/model**	**Model parameters**	**Response delay/time steps**	***N***	**Correct**	**Semantic**	**Formal**	**Mixed**	**Unrelated**	**Non-word**	**RMSD**
SL21		1-s delay	*n* = 89	0.90	0.02	0.00	0.01	0.00	0.06	
Model	s^1^ = 0.000295	8 time steps		0.91	0.02	0.02	0.01	0.00	0.05	*0.010*
SL21	p^2^ = 0.00029	5-s delay	*n* = 85	0.78	0.01	0.00	0.03	0.00	0.11	
Model	DR^3^ = 0.055	25 time steps		0.77	0.06	0.04	0.02	0.01	0.12	*0.026*

UN29		1-s delay	*n* = 90	0.10	0.00	0.07	0.00	0.06	0.77	
Model	s = 0.0003	8 time steps		0.10	0.06	0.11	0.01	0.10	0.63	*0.067*
UN29	p = 0.0003	5-s delay	*n* = 88	0.02	0.03	0.12	0.00	0.22	0.60	
Model	DR = 0.35	25 time steps		0.04	0.04	0.08	0.01	0.08	0.75	*0.084*

DS68		1-s delay	*n* = 90	0.77	0.08	0.02	0.00	0.00	0.13	
Model	s = 0.00024	8 time steps		0.82	0.04	0.03	0.01	0.01	0.09	*0.032*
DS68	p = 0.00022	5-s delay	*n* = 90	0.53	0.18	0.00	0.00	0.00	0.29	
Model	DR = 0.085	25 time steps		0.45	0.08	0.09	0.02	0.05	0.31	*0.070*

*^1^s = semantic weight parameter. ^2^p = phonological weight parameter. ^3^DR = decay rate parameter.*

### Modeling the Pattern of Naming That Shows No Change in Accuracy After a 5 s Response Delay

**FIGURE 2:**
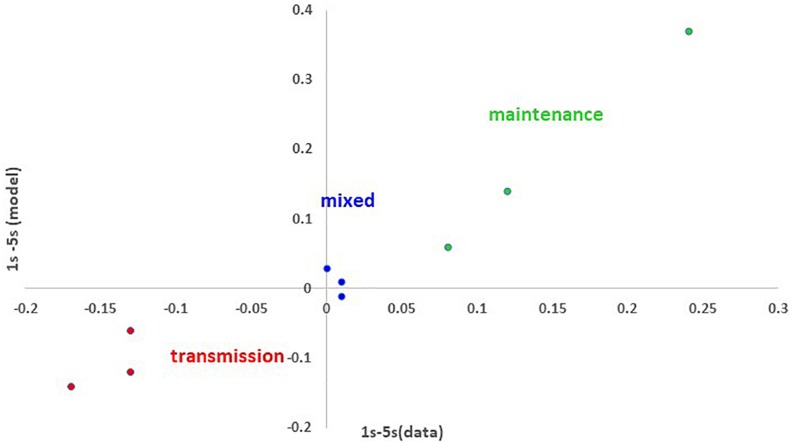
The effects of delay on proportion of correct responses in naming (proportion correct for 1 s minus proportion correct for 5 s) and the model’s fit to this effect for the 9 modeled cases: 3 cases fit with a reduced connection weights (red), 3 fit with increased decay rate (green) and 3 fit with mixed (blue) impairments. The close match between the model and the data is illustrated by the fact that the points fall along a line in which the modeled and actual differences are the same. The fact that the model characterizes negative values (worse performance at 1 s) with change to the weight parameters and positive values (worse performance at 5 s) with changes to the decay parameter, supports the claim that the different effects of delay map onto deficits of transmission and maintenance, respectively.

Thus, far, the slow SP model accounts for those error patterns that become worse or better after a response delay. Can the model account for naming patterns that show no change after a 5 s response delay? We suspect that naming performance is not affected substantially by a 5 s response delay for many if not most people with aphasia. This was true for the sample. There are two types of individuals for whom delay matters little (according to the model). First, there are individuals whose performance is generally very good (e.g., with normal parameters, delay has little effect, see Table 4). Second, those individuals whose lesions include both reduced weights and increased decay are not particularly worse or better after a 5 s response delay, even if their overall level of accuracy is reduced. Table 8 shows three examples of such cases, EC25, HI28, and KM38. The slow SP-decay model fit the data pattern with a lesion in connection weights as well as decay rate. Importantly, the predicted error patterns were unaffected by whether 1 or 5 s of model time had passed. Figure 2 summarizes the modeling of all nine cases. Fits with reduced decay rates simulated a loss in accuracy after 5 s, while fits with reduced connection weights simulated a gain in accuracy. Fits with both lesion types (mixed lesions) simulated three example cases with little change in accuracy as a function of time.

**TABLE 8:** Modeling the pattern of no change in accuracy after a response delay.

				**Response types**						
					
**Participant/model**	**Model parameters**	**Response delay/time steps**	***N***	**Correct**	**Semantic**	**Formal**	**Mixed**	**Unrelated**	**Non-word**	**RMSD**
EC25		1-s delay	*n* = 90	0.68	0.11	0.03	0.03	0.00	0.14	
Model	s^1^ = 0.000145	8 time steps		0.70	0.05	0.06	0.02	0.02	0.15	*0.030*
EC25	p^2^ = 0.00016	5-s delay	*n* = 90	0.68	0.19	0.00	0.00	0.00	0.13	
Model	DR^3^ = 0.04	25 time steps		0.67	0.08	0.06	0.02	0.03	0.15	*0.055*

HI28		1-s delay	*n* = 90	0.81	0.01	0.01	0.00	0.00	0.17	
Model	s = 0.00039	8 time steps		0.77	0.01	0.05	0.01	0	0.17	*0.023*
HI28	p = 0.00015	5-s delay	*n* = 90	0.80	0.01	0.01	0.00	0.00	0.18	
Model	DR = 0.03	25 time steps		0.78	0.04	0.04	0.01	0	0.13	*0.028*

KM38		1-s delay	*n* = 82	0.71	0.12	0.00	0.00	0.00	0.16	
Model	s = 0.00013	8 time steps		0.72	0.07	0.06	0.02	0.03	0.11	*0.040*
KM38	p = 0.00018	5-s delay	*n* = 84	0.70	0.18	0.02	0.02	0.02	0.06	
Model	DR = 0.035	25 time steps		0.71	0.08	0.05	0.02	0.03	0.11	*0.048*

*^1^s = semantic weight parameter. ^2^p = phonological weight parameter. ^3^DR = decay rate parameter.*

## Discussion

In this study, we aimed to provide evidence for word retrieval impairments that arise from impaired activation transmission and/or activation maintenance. This aim is motivated by a model of word processing (Dell et al., 1997) that postulates activation parameters of connection strength and decay rate, that regulate the retrieval and short-term maintenance of lexical-semantic and phonological representations of words. Each parameter affects the success of word retrieval in a different way. Impaired connection weights slow down activation transmission between semantic, lexical, and phonological levels. The s and p connection weights differentially impact semantic-lexical transmission and lexical-phonological transmission, respectively. Impaired decay rate leads to excessive loss of activation by all units at all levels. Another way to think about it is that activation transmission, regulated by the s and p connection weight parameters, reflects how activated units change the activations of other units, while the decay rate parameter determines how a unit’s activation changes regardless of its inputs from other units.

We used a picture naming task with two different response delays to identify differences in transmission and maintenance abilities by persons with aphasia, and we sought to characterize such differences with the model. The model was able to account for the three patterns of change in naming accuracy as a function of delay that we observed: increased accuracy over a delay, decreased accuracy over a delay and little change in accuracy. Improvement in naming accuracy after a delay was modeled with a reduction of semantic and phonological weights, while keeping the decay rate parameter close to a level that simulates accurate word retrieval. To account for the naming pattern of decreased accuracy after a 5 s response delay, it was necessary to make decay rate a lesionable parameter separate from the connection weight parameter. In early simulations of naming and repetition in aphasia (Martin et al., 1996; Dell et al., 1997), decay rate and connection weight were lesioned globally (i.e., throughout the semantic-lexical-phonological network). More recent computational accounts of aphasia using this model lesioned only connection weights, but separately for lexical-semantic connections and lexical-phonological connections. The identification of individuals whose naming accuracy declined following a response delay, necessitated modifying the SP model to allow lesioning of decay rate. In this way, the slow SP model is a more complex model (more lesionable parameters; 3 instead of 2) than the earlier models. But it is also accounting for twice as much data (changes in error patterns as a function of delay) as the original models, thus more than making up for its additional complexity.

Finally, it is important to consider that many individuals in this study were not affected very much by the delay. These can be fit by the model with mixed lesions, that is, with lesions affecting both decay and connection strengths. If it is assumed that within the population of persons with aphasia, the parameters are largely independent random variables, one would expect that most individuals would be in this mixed category. We know from the large modeling study of Dell et al. (2013) that the s and p parameters are completely independent in a group of 103 persons with aphasia. If the same is true for decay with respect to the other parameters, then the relative uncommonness of the “pure” transmission and maintenance deficits that we found is expected.

The original SP model can also simulate word and non-word repetition (e.g., Dell et al., 2007; Nozari et al., 2010). The model assumes that words are repeated by activating a representation of the input and transmitting this activation directly to output phonology (non-lexical route) and indirectly to output phonology via lexical nodes (lexical route). Some patients use both routes while others appear to only use the lexical route (see Nozari and Dell, 2013). We could approach the simulation of word repetition after 1 or 5 s delays in the same way that we have done for naming, that is, by allowing time to pass in the model. For example, participant DS68’s naming was characterized by slow SP-decay model in terms of a decay lesion (Table 7). Using DS68’s parameters derived from naming, we can predict the participant’s word repetition (assuming repetition by just the lexical route) by transmitting activation to the lexical nodes and having that activation spread to the phonology, subject to the altered decay rate. And, using the slow model’s ability to simulate time, we can predict how repetition will be affected by delay. Specifically, DS68’s word repetition is predicted to be 94% correct at a 1 s delay and 65% correct at a 5 s delay. We mention this case because we actually have some data from DS68’s on a word repetition subtest from the TALSA battery (*n* = 45 items). DS68’s performance on this test, which assesses repetition after a 1 and 5 s response delay was quite similar to the model’s predicted performance: 87% correct after a 1 s delay and 58% after a 5 s delay. Thus, the assumed decay impairment derived from the naming data was mirrored in repetition and accurately modeled. Although this is just one case, it exemplifies predictions about repetition that can be made and tested. We stress, though, that success in applying the model to repetition, and more generally to the many phenomena that the original model was applied to over the years, is uncertain. The slow version of the model is not the “same” model as the original version. At this point, we are only confident that the model can explain the naming performance changes with delay, and we are reasonably confident that variation in the slow model’s s and p parameters affects lexical and non-lexical errors similarly as in the original model. For example, lower values of p promote non-word errors.

## Conclusion

When aphasia was first characterized in the 19^th^ century, the focus was on tasks, for example, naming being impaired while repetition is not. Later in the 20^th^ century, theorists described aphasia in terms of impairments to components of linguistic knowledge (e.g., semantics, syntax, phonology) that are necessary to perform those tasks. More recent accounts have emphasized that aphasia is primarily a processing impairment affecting access to linguistic representations rather than a loss of language knowledge (e.g., McNeil, 1982; McNeil and Pratt, 2001). The most recent perspective has sought to characterize the nature of those processing impairments. The goal is not just to say what representations are impaired, but the nature of the impairment. This motivates an emphasis on cognitive abilities such as short-term memory (Saffran, 1990; Martin et al., 1994; Martin and Saffran, 1997), working memory (Wright and Shisler, 2005; Wright and Fergadiotis, 2012; Majerus, 2018), attention (Tseng et al., 1993; Murray et al., 1998; Hula and McNeil, 2008; Martin and Allen, 2012) and executive functions (Miyake et al., 2000; Martin and Allen, 2008; Allen et al., 2012). Thus, a theory of aphasia is evolving to encompass both representational and processing components.

As the theoretical models of aphasia include both linguistic and cognitive aspects of language function, it is anticipated that our approaches to rehabilitation of aphasia will follow suit For example, current assessments of aphasia are able to identify the linguistic stages of word retrieval (semantic and/or phonological) that are impaired, guiding the focus of treatments to one linguistic stage or another (e.g., semantic feature analysis, Boyle and Coelho, 1995; phonological components treatment, Leonard et al., 2008). Our research builds on evidence for two cognitive processes that support word retrieval, transmission and maintenance of activation, demonstrating that impairment to each has differential effects on the time course of word retrieval. As these contrasting impairments become better understood, treatments for anomia potentially will incorporate methods for their remediation. In fact, some treatments are beginning to consider the temporal aspects of interventions (e.g., Kalinyak-Fliszar et al., 2011; Conroy et al., 2018). Although we do not have a theory of how the deficits that we have investigated should be treated, we suggest that varying the temporal demands of responding when pictures and words are trained may be a useful tool, one that may work differently for individuals with maintenance and transmission impairments.

## Data Availability Statement

The datasets generated for this study are available on request to the corresponding author.

## Ethics Statement

The studies involving human participants were reviewed and approved by the Institutional Review Board, Temple University, Philadelphia, PA, United States. The patients/participants provided their written informed consent to participate in this study.

## Author Contributions

Both authors contributed equally to the content of this research. NM provided data and expertise in the assessment, evaluation and interpretation of performances by people with and without aphasia who participated in the behavioral studies. GD directed the computational modeling study and the development of the SP model used in these studies. GD and NM worked together on the model fits to the naming data. Both authors contributed to the interpretation of the results.

## Conflict of Interest

NM and GD confirm that this submitted work was carried out without any personal, professional, or financial relationships present that could potentially be construed as a conflict of interest.

## Footnotes

1 We emphasize that the slow model is not a response-time model in the way that other production models are (e.g., Levelt et al., 1999; Oppenheim et al., 2010; Roelofs, 2014). It has no mechanism for varying selection time as a function of activation.
